# All Sustainable Development Goals Support Good Health and Well-Being

**DOI:** 10.3389/ijph.2023.1606901

**Published:** 2023-12-27

**Authors:** William B. Weeks, James N. Weinstein, Juan M. Lavista

**Affiliations:** ^1^ AI for Good Lab, Microsoft Corporation, Redmond, WA, United States; ^2^ Microsoft Research, Microsoft Corporation, Redmond, WA, United States

**Keywords:** public health, sustainability, sustainable development goals, well-being, good health

Margaret Mead said that the first sign of civilization was a broken femur that had been healed. Humanity’s willingness and ability to care for one another defines civilization; without that inclination, we are considered animals.

Since civilization began, humanity has become more sophisticated in caring for itself. Now, we attempt to fix broken hearts, psyches, and appendices as well as femurs. Although we have different ways of funding those fixes, there is a general global sense that health and healthcare are fundamental rights. In the United States, our founding fathers declared a right to life, liberty, and the pursuit of happiness: while life and health may not be equivalent, the absence of the former eradicates the latter, and the absence of the latter likely compromises liberty and the pursuit of happiness.

**FIGURE 1:**
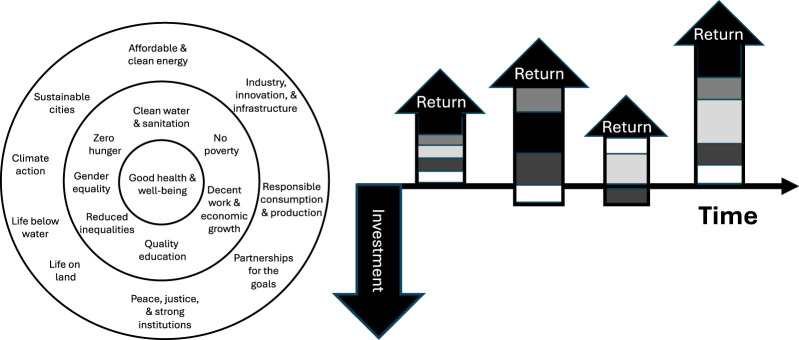
A proposed relationship between Good Health and Well-Being (SDG 3) and the other 16 SDGs, based on Bronfenbrenner’s Ecological Systems Theory (left), and a proposed method for evaluating returns on investment in the other SDGs to Good Health and Well-Being (right), where different shades indicate different measures of good health and well-being.

In civilization’s evolutionary development, humanity has done a lot of harm to its members and the planet. To redress that harm, in 2015, the United Nations General Assembly (UNGA) defined 17 sustainable development goals (SDGs, listed in the Figure 1) to serve as a “shared blueprint for peace and prosperity for people and the planet” that were adopted into the UNGA’s 2030 Agenda, when most goals are to be achieved. Sustainable Development goal 3, (SDG3) is, “Good Health and Well-Being.”

The Global Burden of Disease Collaboration has created a way to track progress on SDG 3 by creating an index of 37 health-related indicators that have been calculated for 1990 through 2030. The effort has uncovered some progress, but much room for improvement: only 5% of countries are estimated to achieve SDG goals by 2030, and then on only 11 of 17 the indicators [1].

While aggregating health-related measures to estimate local health and wellbeing makes sense, the reality is that all SDGs are related to health. The World Health Organization has defined the social determinants of health (SDOH) as “the conditions in which people are born, grow, work, live and age, and the wider set of forces and systems shaping the conditions of daily life.” [2] With evidence that SDOH have more impact on health than does clinical care, and that many interventions designed to address health inequities are SDOH-focused [3], it is important to understand the relationship between Good Health and Well-Being and the other SDGs.

We provide a representation of that relationship on the left side of the Figure 1. Based on Bronfenbrenner’s Ecological Systems Theory, wherein human development is influenced by different environmental systems [4], so human health and wellbeing is influenced by the multiple SDG-defined systems in which human lives are immersed. It is hard to imagine achieving Good Health and Well-Being in a world of hunger, poverty, and lack of sanitation; it is hard to imagine achieving a hunger-less, poverty-less, and sanitary world in the absence of responsible consumption, peace and justice, and sustainable environments. In both examples, the central goal is embedded in supporting goals without which the central goal cannot be achieved.

We also propose a different way to evaluate investments in SDG efforts. Just as with efforts to disaggregate health into a multiplicity of measures to understand progress on achievement of Good Health and Well-Being, evaluation of progress toward attaining zero hunger or responsible consumption and production or affordable and clean energy might be disaggregated into component measures (like reduced subjective hunger, reduced malnourishment, ample food stocks; less waste, more recycling, more efficient production; and less reliance on fossil fuels, less carbon release, and relative decline in energy costs, respectively). On the right side of the Figure 1, we propose that investments in each of the other SDGs be evaluated, in part, on that investment’s return to disaggregated or aggregated measures of Good Health and Well-Being [1]. As with any investment, returns are likely to vary in magnitude over time, can possibly be negative, and differ in the form in which they accrue. For example, returns to measures of wellbeing might differ in magnitude and over time from those to overall health status or decreases in respiratory disease.

Our proposed investment framework has an advantage: it is more understandable and less abstract than changes in measures of carbon emission, particulate matter, or water quality. Human behavior change is required to achieve the supporting SDGs. Humans might be more motivated to change their behaviors if the goal is expressed in terms of ultimate outcomes—lives saved or improved longevity—than in more abstract or unrelatable measures of outputs—carbon emissions or water quality. Further, the overarching goal of Good Health and Well-Being can be used to drive policies designed to meet SDG goals: since the 1970s, Bhutan has prioritized happiness over gross domestic product, pursuing policies designed to maximize its gross national happiness, measured as an index built on the pillars of good governance, sustainable and equitable socioeconomic development, cultural preservation, and environmental conservation [5].

Adam Smith, who understood the economic impact of the industrial revolution, and Thomas Malthus, who fretted about the population growth that the industrial revolution fueled, were moral philosophers who understood human behavior and thought about the long-term social consequences of economic growth. Just as technological advancement was the engine of that growth and the solution to Malthus’ concern, it is needed to address climate change and achieve the SDGs that support Good Health and Well-Being. Those technological advancements will require investments; returns on those investments should be measured as improvement in Good Health and Well-Being.

Reframing the SDGs to highlight the centrality of achieving Good Health and Well-Being motivates human behavior change. Just as with the first one, the next iteration of civilization will emphasize the pursuit of Good Health and Well-Being; however, in this modern world, where man has become the master of his environment and the mechanism for changing it, the role of the other SDGs should be understood as support for that primary pursuit, and they should be considered and evaluated as adjunctive to it.
